# Green synthesis of silver nanoparticles using sumac fruit by microwave and traditional methods: characterization, anticancer, and antimicrobial activities

**DOI:** 10.55730/1300-0527.3751

**Published:** 2025-07-21

**Authors:** Melek HINIS, Tuğçe KARADUMAN YEŞİLDAL, Demet ERDÖNMEZ, Ayfer MENTEŞ

**Affiliations:** 1 Scientific and Technological Application and Research Centre, Aksaray University, Aksaray, Turkiye; 2 Department of Molecular Biology and Genetics, Faculty of Science and Letters, Aksaray University, Aksaray, Turkiye; 3 Department of Pharmaceutical Microbiology, Faculty of Pharmacy, Düzce University, Düzce, Turkiye; 4 Department of Chemistry, Faculty of Science and Letters, Aksaray University, Aksaray, Turkiye

**Keywords:** Antimicrobial, anticancer, green synthesis, silver, sumac, microwave-assisted synthesis

## Abstract

Green synthesis of silver nanoparticles (Ag-NPs) was carried out with sumac fruit extract using a microwave-assisted (MWA) method and a traditional method (TDM). The properties of nanoparticles synthesized by both methods were characterized and compared.Although both methods are environmentally friendly, the MWA method was faster, more efficient, and economical.

When creating Ag-NPs, variables like temperature, pH, reaction duration, extract concentration, and silver ion concentration were considered. The production of Ag-NPs was confirmed by ultraviolet–visible spectra that displayed the surface plasmon resonance band centered at 433 and 436 nm in the MWA and TDM techniques, respectively. The results of the scanning electron microscopy/energy-dispersive X-ray spectroscopy analysis indicated that the nanoparticles were spherical in structure and that the amount of Ag was significantly higher than that of other elements. According to transmission electron microscopy analysis, particle sizes were 22 nm with the TDM method, while particle sizes were 41.85 nm with the MWA method. However, the MWA method had more uniformly dispersed and homogeneous particle sizes. Conductivity measurements of Ag-NPs solutions were obtained following each cycle of washing. Subsequent to 3 cycles of washing, the conductivity approached that of deionized water, indicating the effective removal of unreacted ions. In our study, a significant increase was detected in the antibacterial and antifungal activities of Ag-NPs. Furthermore, both Ag-NPs inhibited the proliferation of HT-29 cells and showed a selective anticancer effect against intestinal cancer cells without showing toxicity (all cell viability values >70%) to healthy control L929 fibroblast cells. This study is the first comparative analysis of TDM and MWA methods using sumac for both antimicrobial and anticancer assessment.

## 1. Introduction

Nanoparticles, particles with sizes of 100 nm or less, have been used in electrical, biomedical, medical, and cosmetics sectors in recent years due to their functionality and properties [1,2]. Nanoparticles of various metals and metal oxides have been synthesized using different synthesis techniques [3]. In particular, silver nanoparticles (Ag-NPs) have become important in recent years due to their biological effectiveness, low cost, and low toxicity [2,4].

Silver has antimicrobial properties and has historically been used in the treatment of many diseases and biomedical applications, including eye drops, water disinfection, and the treatment of burns and wounds [5,6]. Ag-NPs synthesized in recent years have attracted attention because of their antibacterial, antifungal, antiviral, anticancer, and antioxidant activities [2,7,8]. It is important to develop materials containing Ag-NPs as an alternative to antibiotics due to their potential to stop infections without causing antibiotic resistance. Due to their unique properties, Ag-NPs are used as antimicrobial agents in a number of sectors, including the food industry, food packaging materials, the textile industry, and the cosmetic industry. They are also utilized in a variety of other areas, including catalysis, sensors, bioapplications, DNA sequencing, climate change, and sedative delivery [1,9].

Many classical physical, chemical, and biological methods have been used for the synthesis of metallic nanoparticles. The most common methods are chemical, with a fast synthesis time. However, they are expensive and toxic chemicals are generally used during the synthesis. Furthermore, unreacted chemicals may be present in the solutions [10]. Therefore, nontoxic, inexpensive, and environmentally friendly green synthesis of nanoparticles is of significant importance. Microorganisms (bacteria, fungi, and algae) are used in the biological synthesis of nanoparticles. In these methods, the synthesis reaction takes a long time (24–124 h), as does the preservation of cell cultures [11]. Thus, the development of synthesis methods with green chemistry has become an important challenge in recent years. With green synthesis, natural extracts obtained from the roots, stems, fruits, and vegetables of plants are used. Carbohydrates, polyphenols, terpenoids, gums, proteins, saponins, sugar, steroids, and flavonoids contained in plant extracts serve as reducing agents during the synthesis of Ag-NPs. Nanoparticles synthesized with this method are environmentally friendly and cost effective and do not require high temperature, pressure, and energy [1,12]. Additionally, these nanoparticles are suitable for biomedical and pharmaceutical applications due to their biocompatibility.

Sumac (*Rhus coriaria* L.) is an endemic plant belonging to the Anacardiaceae family, which has more than 250 species [13]. It grows wild from the Canary Islands to Iran and Afghanistan along the Mediterranean coastline [14]. It grows in the Mediterranean and Southeastern Anatolia regions of Türkiye. Sumac is often used as a spice in meals. The fruit extracts contain organic acids (such as malic, citric, and tartaric acid), hydrolyzable tannins, flavonoids, anthocyanins, saponins, phenolics, and volatile compounds [15,16]. Studies have shown that sumac fruit is biologically active and has antioxidant, antimicrobial, antiobesity, and antidiabetic activities [17–21]. Owing to antioxidants such as tannin and phenolic compounds settled on the surface of nanoparticles synthesized with sumac, these nanoparticles can be used as anticarcinogenic agents with a surface effect in their applications [22].

Traditional methods (TDMs) have mostly been used in the synthesis of Ag-NPs, which are generally performed using plant extracts [23–25]. The microwave-assisted (MWA) method has been preferred in recent years because it is faster, more energy efficient, and more economical than the TDMs [26]. The aim of this study was to compare the physicochemical properties and biological (antimicrobial and anticancer) effects of Ag-NPs synthesized by the TDM and the microwave method. Green synthesis of Ag-NPs was carried out using sumac fruit extract. Obtaining the extract and synthesis of nanoparticles was carried out by both methods.

## 2. Materials and methods

### 2.1 Materials

Silver nitrate (AgNO_3_) and sodium hydroxide (NaOH) were purchased from Merck Millipore (Burlington, MA, USA). All reagents were analytical grade and used without further purification. The sumac plants (*R*. *coriaria*) were harvested in September 2023 from Naltaş Village in Adana Saimbeyli district in Türkiye (384′31″N, 363′4″E). The sumac plants were collected and cleaned, and their fruits were ground into a fine powder using a grinder. The ground sumac fruit was stored at 4 C.

### 2.2 Green synthesis of Ag-NPs by the TDM

#### 2.2.1 Preparation of sumac fruit extract by the TDM

For the TDM, 5 g of ground sumac fruit was added to 100 mL of distilled water. The solution was stirred at 80 C for 60 min. The extract solution was filtered using a porosity 3 filter after being cooled. The extract was stored at −20 C in a freezer until further use [22].

#### 2.2.2 Green synthesis of Ag-NPs by TDM

At room temperature, 50 mL of 1 mM AgNO_3_ solution was mixed with 15 mL of sumac fruit extract. The reaction was completed after 24 h. The color changed from pink to dark brown. The dark brown color indicated the reduction of ionic silver to metallic Ag-NPs by sumac fruit extract. The Ag-NPs were separated from the solution by centrifuging at 5000 rpm for 35 min and washed with distilled water 3 times. Some of the obtained particles were put in watch glasses and dried at room temperature for characterization. Some of the particles were stored in a falcon tube with distilled water at 4 C for later experiments.

Ag-NPs were synthesized with increasing volumes of sumac extract (SMC) (5, 10, 15, 20, and 25 mL) and different concentrations of AgNO_3_ (1, 2, 3, and 10 mM). To evaluate the synthesis under basic conditions, this synthesis was repeated in a solution with a pH of 9. The pH of the solution was adjusted with 0.1 M NaOH solution. The reaction was completed in 5 min. The results indicated that the pH value of the reaction increased the reaction rate.

### 2.3 Green synthesis of Ag-NPs by the MWA

#### 2.3.1 Preparation of sumac fruit extract by the MWA

For the MWA method, 5 g of ground sumac fruit was added to 100 mL of distilled water. The solution was kept under reflux in microwave heating for 5 min. (800 W, 2450 MHz). The extract was filtered using a porosity 3 filter after being cooled [27]. The extract was stored at −20 C for future use.

#### 2.3.2 Green synthesis of Ag-NPs by MWA

For synthesis, 50 mL of 1 mM AgNO_3_ solution was mixed with 15 mL of MWA sumac fruit extract. The mixture was kept under reflux in a microwave (800 W, 2450 MHz) for 5 min. until a brown color was observed, which indicated the formation of Ag-NPs. The color changed to dark brown when the solution cooled [27]. The solution was centrifuged at 5000 rpm for 35 min. and washed with distilled water 3 times. Some of the obtained particles were put in watch glasses and dried at room temperature for characterization. Some of the particles were stored in a falcon tube with distilled water at 4 C for later experiments.

### 2.4 Characterization of nanoparticles

Ultraviolet-visible (UV–Vis) spectra of the samples were measured by surface plasmon resonance (SPR) morphology with a 1 cm quartz cell between 300 and 800 nm wavelengths using Genesys 10S UV–Vis Spectrophotometer (Thermo Fisher Scientific, Waltham, MA, USA). Fourier transform-infrared spectrometry (FT-IR) (4000–650 cm^−1^) was recorded on a Perkin Elmer Spectrum 100 FT-IR Spectrophotometer (Waltham, MA, USA) using the attenuated total reflectance technique. Transmission electron microscopy (TEM) was used to examine the morphology and size of Ag-NPs. Images of Ag-NPs were obtained from an FEI TALOS F200S TEM microscope (Thermo Fisher Scientific) operating at 200 kV. The size distribution of the particles was determined with ImageJ software. Ag-NPs were characterized by scanning electron microscopy (SEM) using a Quanta FEG 250 (Thermo Fisher Scientific). Elemental analysis of nanoparticles was carried out with energy-dispersive X-ray (EDX) spectrometers. The crystalline structure of nanoparticles was measured by X-ray diffraction (XRD) with a Malvern Panalytical Empyrean X-ray diffractometer (Malvern, UK). The conductivity and pH of solutions were measured at 25 C with a table top CD-2005 conductometer (JP Selecta, Barcelona, Spain). The amount of silver in the synthesized nanoparticles was determined by an X Series 2 inductively coupled plasma mass spectrometry (ICP-MS) instrument (Thermo Fisher Scientific).

### 2.5 In vitro antimicrobial activity assay

The microorganisms *Staphylococcus aureus* ATCC 29213, *Streptococcus pneumoniae* ATCC 49619, *Escherichia coli* ATCC 25922, *Pseudomonas aeruginosa* ATCC 27853, *Enterococcus faecalis* ATCC 29212, and *Candida albicans* ATCC 10231 were resuspended from stock using brain heart infusion broth. Mueller–Hinton agar was used for agar well diffusion assays. An agar corkborer was used to create a hole of the desired diameter in the agar; after each use, it was sterilized in alcohol followed by flaming. To culturing microorganisms, 0.5 McFarland (1.5 × 10^8^ CFU/mL) bacterial solution was prepared from the overnight culture and smeared onto agar plates using a swab [28,29]. Standard antimicrobial drugs (ciprofloxacin for bacteria and fluconazole for yeast) were screened under similar conditions for comparison. Activity was determined at the end of 24 h by measuring the diameter (in millimeters) of the zone showing complete inhibition, and the experiments were performed in 3 replications [30]. The compounds tested for antibacterial and antifungal activities were dissolved in DMSO and diluted to a final DMSO concentration of ≤1% (v/v) in all wells to ensure no solvent-related effects. The initial concentration of 100 mg/mL was prepared in the first well of the series for each microorganism, followed by 6 dilutions (100, 50, 25, 12.5, 6.25, and 3.12 mg/mL). Control wells containing only DMSO (≤1% v/v) without test compounds or microorganisms were included to account for any solvent effects. The density of the 18- to 24-hour broth cultures of bacteria and yeast were standardized before use.

In addition, control wells containing only the compounds and no microorganisms were prepared. All microplates were incubated at 37 C for 24 h. The lowest dilution in the tube that showed no turbidity (i.e. no growth) was determined as the minimum inhibitory concentration (MIC/MBC) in milligrams per milliliters (mg/mL) [31].

### 2.6 Cell culture studies

The anticancer and cytotoxic activity of the Ag-NPs was evaluated using HT-29 and L929 cell lines. The human colon cancer cell line HT-29 (HUKUK, Şap Institute, Ankara, Türkiye) and the healthy mouse fibroblast line L929 (HUKUK, Şap Institute, Ankara, Türkiye) were cultured in high-glucose DMEM medium (Sigma-Aldrich, St. Louis, MO, USA, catalog number: D6429) containing 10% fetal bovine serum and 1% penicillin/streptomycin. Cells were checked microscopically and passaged with trypsin when they reached 70% confluency [32,33].

The samples were initially dissolved in DMSO and prepared at stock concentrations, with a sonicator used to ensure particle dispersion. These stock solutions were subsequently diluted with DMEM medium to achieve final concentrations ranging from 6.25 to 100 μg/mL. The final concentration of DMSO in all treatment and control wells was adjusted to be equal and kept at 0.1% to eliminate solvent-related effects. DMSO-only control wells were also included to assess any potential influence of the solvent. 5-fluorouracil (5-FU) at a concentration of 50 μg/mL has been used as a positive control in anticancer studies conducted on HT-29 cells.

Cell viability was determined using 3-(4,5-dimethylthiazol-2-yl)-2,5-diphenyltetrazolium bromide (MTT). This approach measures mitochondrial dehydrogenase activity in cells. For the assay, both cell lines were seeded into 96-well plates at a density of 1 × 10^4^ cells per well and incubated at 37 C with 5% CO_2_. After 24 h, the cell culture medium was replaced with a fresh medium containing the relevant nanoparticles at concentrations ranging from 6.25 to 100 μg/mL. The medium in the control wells was also refreshed. At the end of the 48-hour incubation period, the MTT solution was added to each well to a final concentration of 0.5 mg/mL. After 3 h of incubation, the formation of formazan crystals was observed, and the crystals were dissolved in DMSO. The resulting color was measured spectrophotometrically at 492 nm using a ChroMate ELISA reader (Awareness Technologies Inc., Palm City, FL, USA). Each experiment was performed in triplicate and conducted in parallel for both cell lines Wells containing only the culture medium, without extract, were used as the control group. The percentage of cell viability was determined relative to the control group using the following formula, with cell viability in the control groups accepted as 100%.


(1) % Viability=[(A sample)/(A control)]×100

Where a control represents the absorbance of the control well, and A sample represents the absorbance of the extract application well. IC_50_ values were determined using GraphPad Prism Version 5 (San Diego, CA, USA), and cytotoxicity evaluation was performed on healthy control cells (L929) at the dose concentration range including this IC_50_ value.

### 2.7 Statistical analysis

The data analysis was performed using GraphPad Prism version 5 software (San Diego, CA, USA). The data represent the mean and standard error (SE) of the mean. Statistical differences were evaluated using 2-way ANOVA followed by the Bonferroni posthoc test (95% confidence interval). P-values less than 0.05 were considered statistically significant (* = p < 0.05, ** = p < 0.01).

## 3. Results and discussion

**Figure 1:**
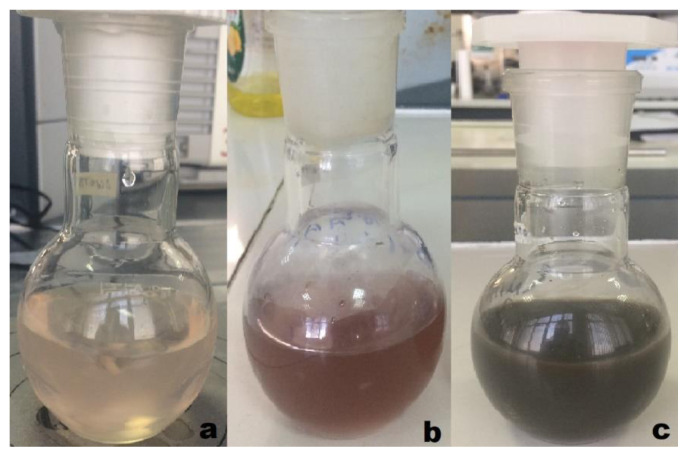
Synthesis of TDM Ag-NPs using SMC (a) initially, (b) at 3 to 3.5 h, and (c) at the end of the reaction (24 h).

The first indicator of Ag-NP formation in TDM synthesis was the color change in the solution. The color of the SMC and AgNO_3_ solution for TDM was pale pink at the beginning of the reaction. Over time (3–3.5 h), the solution turned light brown. At the end of the reaction (24 h), it turned completely dark brown (Figure 1).

**Figure 2:**
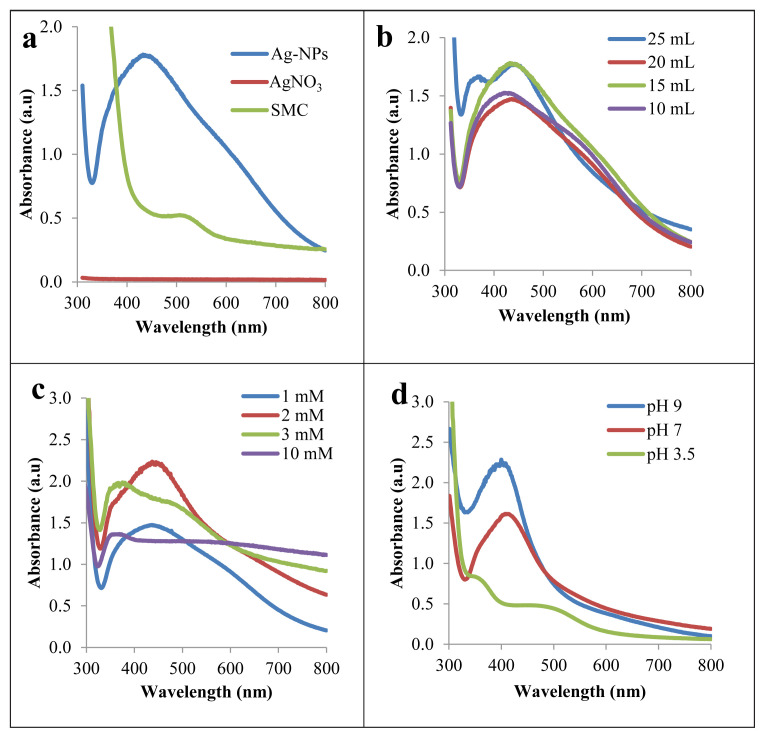
(a) UV–Vis absorption spectra of Ag-NPs synthesized using SMC, (b) 1 mM AgNO_3_ solution and different volumes of SMC, (c) 15 mL SMC and different concentrations of AgNO_3_, and (d) Ag-NPs spectra at different pH values.

UV–Vis spectroscopy is one of the most widely used methods for the characterization of synthesized Ag-NPs. Ag-NPs formation was analyzed by UV–Vis spectrophotometer with measurements from 300 to 800 nm wavelengths. Ag-NPs absorption spectra were observed by SPR bands at 436 nm and 433 nm for TDM and MWA, respectively. These are the expected SPR characteristics of Ag-NPs [34,35]. The literature has shown that the optical density of the SPR peak increases with an increase of nanoparticles in the reaction mixture. The sharpness of this peak also indicates the relative homogeneity of the size distribution of the synthesized particles [36]. In the TDM Ag-NPs, no initial color change was observed at all concentrations. After 3.5 h, the color started to change. The reaction was completed after 24 h. Figure 2a shows the SPR formation that occurs during Ag-NPs synthesis with 50 mL of 1 mM AgNO_3_ and 15 mL of SMC in 24 h. The absorption band of Ag-NPs is clear evidence of Ag-NP formation when compared with the SMC and AgNO_3_ solutions.

To optimize Ag-NP synthesis, experiments were repeated using different volumes of SMC and different concentrations of AgNO_3_ solution.

Plasma resonance peaks of nanoparticle synthesis with 50 mL of 1 mM AgNO_3_ solution and different volumes of SMC (10, 15, 20, and 25 mL) are shown in Figure 2b. The sharpest and most intense peak was observed when using 15 mL of SMC. The 20 mL volume of SMC produced a lower absorption peak. The higher concentration of the SMC caused the Ag^+^ particles to decrease very quickly and aggregate. The rapid formation and aggregation of Ag-NPs in solution indicated the formation of multiple overlapping absorption peaks [36]. The observed spectrum at 25 mL of SMC showed that the nanoparticles formed were not pure, as 2 separate peaks were observed at both 430 nm and 390 nm. The peak at 390 indicates AgCl formation [37,38].

Different concentrations of AgNO_3_ (1, 2, 3, and 10 mM) were used for synthesis with 15 mL SMC. The absorption value increased in 2 mM AgNO_3_ solution (Figure 2c). The absorption bands shift below 390 nm when AgNO_3_ was at 3 and 10 mM concentrations. As indicated in the literature, signals below 390 nm suggest the presence of impurities, organic compounds, or solvent residues in the sample [23]. Compounds found in plant extracts can cause AgCl formation. For this reason, the peaks around 390 nm may be related to the formation of AgCl nanoparticles [37,38].

According to the literature, pH has a strong effect on the formation of nanoparticles. Studies have shown that pH affects the amount and stability of nanoparticle production and is a critical factor in controlling the size and morphology of nanoparticles. Additionally, pH affects the rate of the reduction reaction [27,35].

In our study, to understand the effect of pH on Ag-NPs synthesis, the UV–Vis spectra of samples synthesized at pH 3.5, pH 7, and pH 9 were analyzed. In TDM synthesis at room temperature, the initial pH of the SMC was 3.5, and the solution pH also remained after AgNO_3_ was added. In this method, there was no change in the color of the solution initially. After 3.5 h, the solution color started to change, and the reaction was completed after 24 h. As soon as the pH value of the mixture was adjusted to pH 7 and pH 9 with 0.1 M NaOH, the color started to change immediately. After 1 h, the color completely changed to dark brown. The reaction was completed after 3h. Figure 2d shows the absorption spectra of Ag-NP formation according to pH values at the 3 h. Accordingly, as pH increased, the highest and sharpest absorption peak was observed at pH 9. pH affected the reaction rate and increased the number of nanoparticles formed. At the same time, a monodisperse suspension was formed due to the sharp absorption band formed at pH 9.

**Figure 3:**
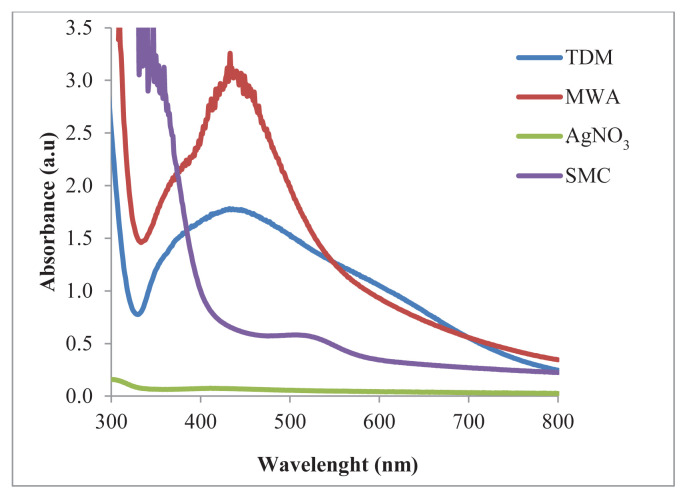
UV–Vis absorption spectrum of Ag-NPs synthesized with SMC.

In MWA synthesis, the initial color of the mixture of SMC and AgNO_3_ solution was light pink, just like in the TDM. The color of the solution started to change after being placed in the microwave for 5 min. After cooling, the color turned brown. In Figure 3, when compared to the absorbance of SMC and AgNO_3_, the formation of Ag-NPs is clearly seen with the formation of the SPR absorption band at 433 nm. Compared to the TDM, the peak obtained in MWA synthesis is sharper, and the absorbance value is higher. This means that the number of nanoparticles synthesized with MWA synthesis is higher and has a more homogeneous distribution [39,40].

**Figure 4:**
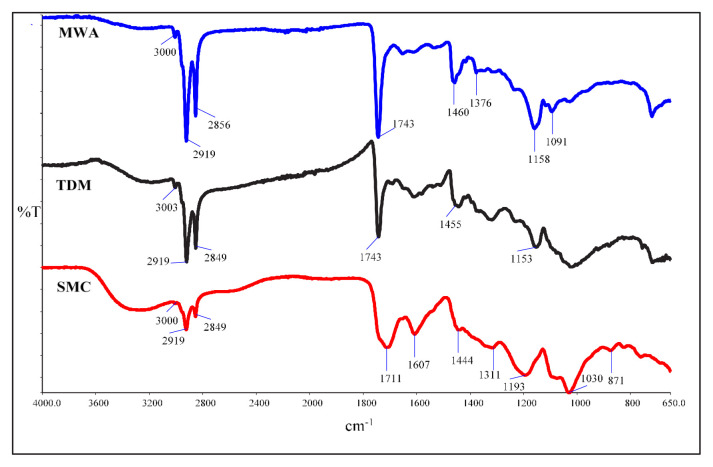
FT-IR spectra of SMC, TDM Ag-NPs, and MWA Ag-NPs.

Figure 4 shows the FT-IR spectra of SMC and Ag-NPs synthesized by TDM and MWA methods. The broad peak at about 3200 cm^−1^ in SMC corresponds to the O–H stretching of functional groups such as alcohol, carboxylic acid, and carbohydrates. The peak at 3000 cm^−1^ corresponds to aromatic groups, and the peaks at 2919–2849 cm^−1^ correspond to the vibration of aliphatic −CH and −CH_2_ groups. The peaks at 1711 and 1607 cm^−1^ correspond to the carbonyl C=O and C=C alkene stretching bands, respectively. The O–H bending vibration of phenolic groups is observed at 1311–1193 cm^−1^. Strong peaks at 1030 cm^−1^ showed the C–O stretching of the primary alcohol bond. The peaks were specific for the tannins and flavonoids found in the structure of SMC [17,27]. Functional groups belonging to biomolecules such as flavonoids and tannins, which are responsible for the reduction of Ag ions, were detected in the FT-IR spectrum.

When the spectra of the synthesized Ag-NPs were compared, the broad −OH peak at 3200 cm^−1^ in the SMC is weaker in the TDM and MWA methods. The change in this is indicative of Ag^+^ ions being reduced to Ag nanoparticles. In the spectrum, the −CH and −CH_2_ bands were sharper at 2919 cm^−1^ and 2849 cm^−1^, respectively. The peaks observed at 1711 cm^−1^ and 1607 cm^−1^ in the SMC changed, and the strong carbonyl peak at 1743 cm^−1^ showed that the binding occurred through the C=O group after the formation of Ag-NPs. Similarly, there were shifts in the peaks around 1312, 1193 cm^−1^, and 1030 cm^−1^. The formation of new peaks at 1153 cm^−1^ and 1158 cm^−1^ in TDM and MWA, respectively, indicates the formation of new bonds with functional groups in the SMC [22].

**Figure 5:**
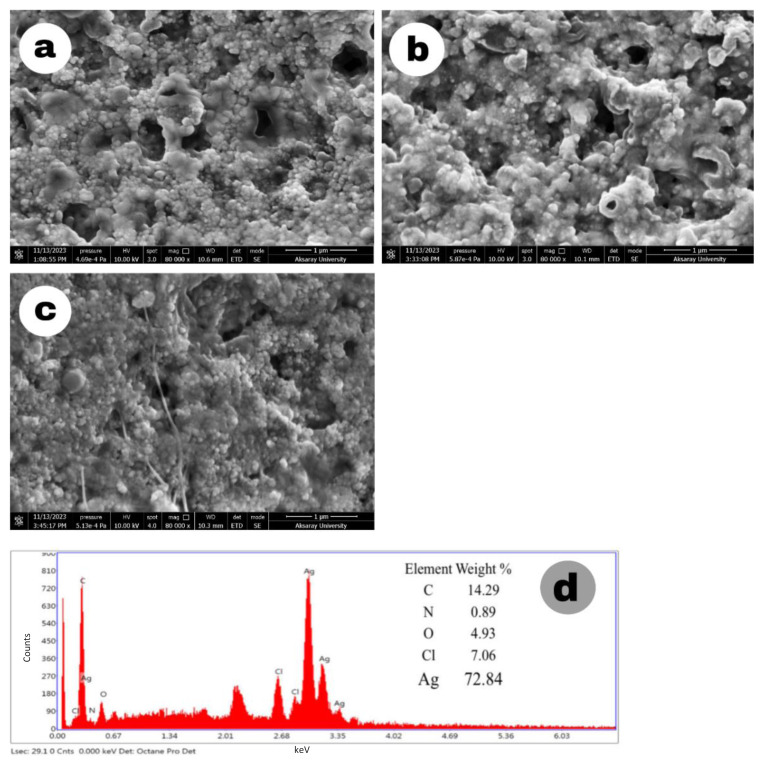
SEM images of Ag-NPs synthesized with 1 mM AgNO_3_ and (a) 15 mL, (b) 20 mL, and (c) 25 mL of SMC; and (d) the EDX spectrum with 15 mL SMC.

The morphological structures of Ag-NPs synthesized under different conditions were examined by SEM/EDX. Images of Ag-NPs synthesized by TDM with 15, 20, and 25 mL volumes of SMC with 1 mM AgNO_3_ are shown in Figure 5. In the SEM images taken at 80,000× magnification, Ag-NPs were uniformly structured, spherical, and less than 100 nm.

**Figure 6:**
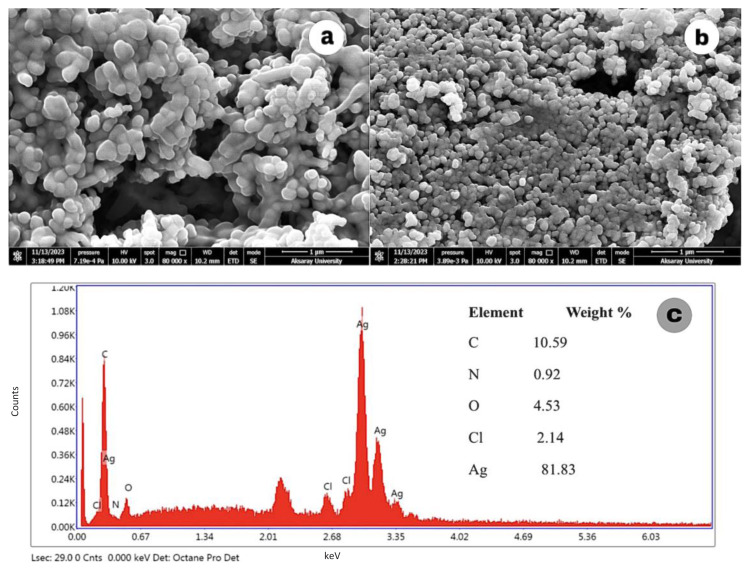
SEM images of Ag-NPs at (a) 40 C, (b) room temperature, and (c) EDX spectrum at room temperature.

**Figure 7:**
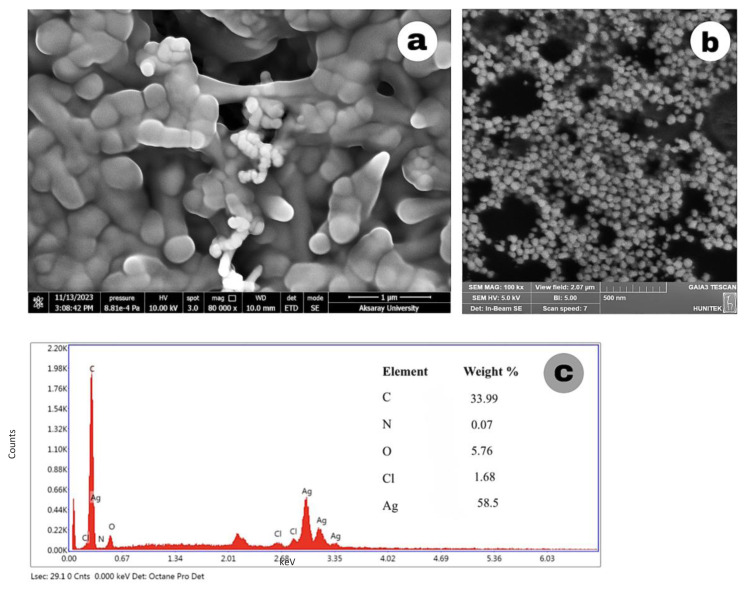
SEM images of Ag-NPs synthesized with (a) 3 mM AgNO_3_ (TDM), (b) 1 mM AgNO_3_ (MWA), and (c) the EDX spectrum of 3 mM Ag-NPs.

Figure 6 shows the images of Ag-NPs synthesized with 2 mM AgNO_3_ at room temperature and 40 C. Ag-NPs synthesized at room temperature had a spherical structure and a rather uniform distribution compared to the nanoparticles synthesized at 40 C. The 3 mM Ag-NPs SEM image in Figure 7a was not clear and the particle structures were not evenly distributed. The Ag signal was weak in the EDX result, indicating that the nanoparticles were not formed as expected. Furthermore, this structure did not show a uniform distribution in the UV spectrum. In Figure 7b, the images of Ag-NPs synthesized by the MWA method show a very uniform distribution and a spherical structure.

Strong Ag signals seen in the EDX analysis of Ag-NPs also confirm the presence of Ag-NPs. Additionally, the presence of weak peaks such as C, N, and O in the EDX data indicates the presence of phytochemicals from SMC.

**Figure 8:**
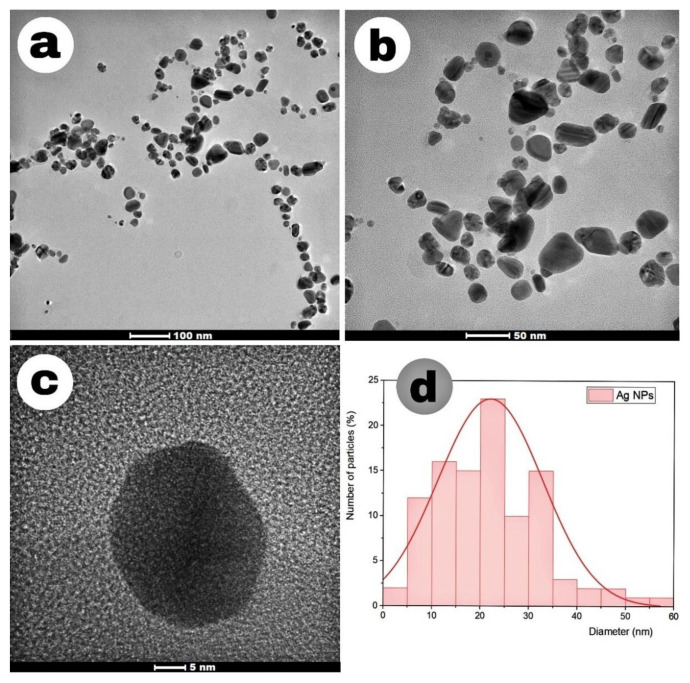
(a–c) TEM images of Ag-NPs (TDM) at different magnifications. (d) Histogram showing the nanoparticle size distribution.

**Figure 9:**
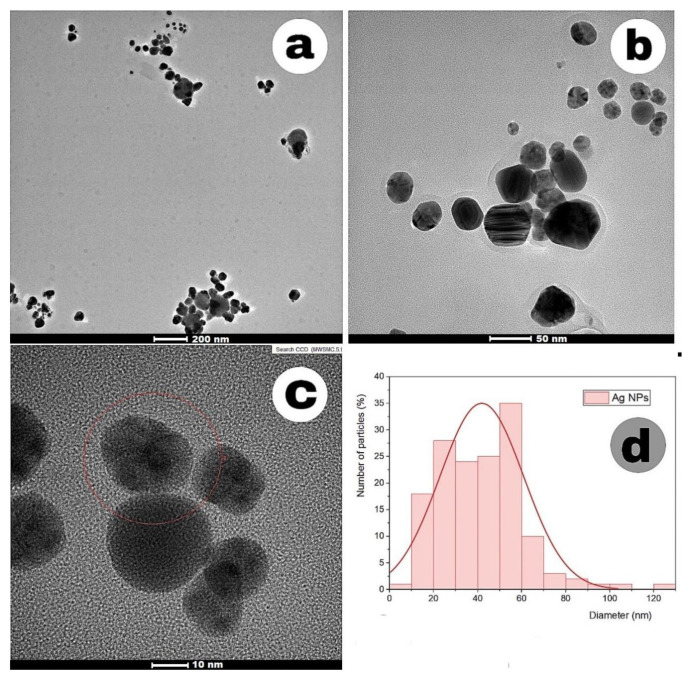
(a–c) TEM images of Ag-NPs (MWA) at different magnifications. (d) Histogram showing the nanoparticle size distribution.

The morphology and size of Ag-NPs were determined by transmission electron microscopy (TEM). Figure 8 and Figure 9 show the TEM images and size distribution diagrams of Ag-NPs synthesized by the TDM method and the MWA method. Most of the synthesized nanoparticles were spherical and well dispersed. Particle distribution histograms show that the particle size of Ag-NPs synthesized by the TDM was 22 nm, while the nanoparticles synthesized by the MWA method were 41.85 nm. The average particle size of the nanoparticles obtained from SMC proved that the particles were of the expected size and well distributed. This shows that the biological agents found in SMC are effective agents in reducing Ag^+^ to Ag^0^.

**Figure 10:**
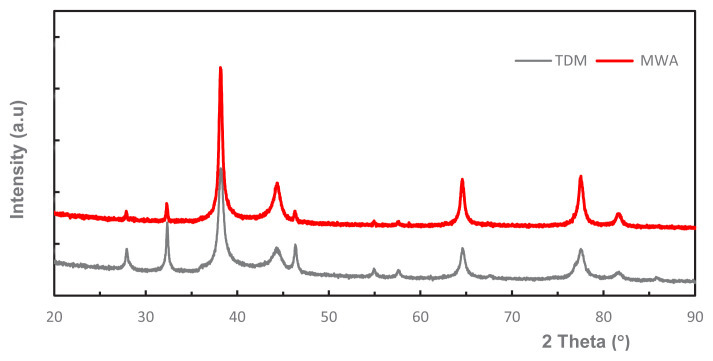
XRD patterns of Ag-NPs (TDM and MWA).

Figure 10 shows the XRD patterns of Ag-NPs synthesized by TDM and MWA methods. Bragg reflection with 2θ values at 38.24, 44.24, 64.64, and 77.52 were observed that are characteristic diffraction peaks corresponding to planes of silver (111), (200), (220), and (311), respectively. The obtained values suggest the crystal structure is face-centered cubic for silver (JCPDS, 4-0783) [2,25]. Additional unidentified peaks were also observed in the diagram at 27.95, 32.34, 46.34, 54.92, and 57.66. These peaks may be attributed to the presence of trace amounts of Ag_2_O or AgCl that are likely to have formed during the reaction. Some Ag-NPs may be converted to silver oxide by oxidizing agents in the extract [41,42]. Similarly, the peaks seen outside the Ag-NPs in the diagram may be AgCl precipitate formed by the reaction of the small amount of AgNO_3_ in the reaction medium with the chlorides coming from the plant extract [23,43–45]. The presence of chloride and oxide ions in the SEM/EDX data suggests that these peaks may result from both Ag_2_O formation and AgCl precipitation.

The average size of the nanoparticles was calculated through Debye–Scherrers formula:


(2) $$D=\frac{k\lambda }{\beta cos\theta }$$

Where D is the average crystalline size of NPs, k is the geometric factor (0.9), λ is the X-ray wavelength, and β is the angular full width at half-maximum of the XRD peak at the diffraction angle θ [46,47]. The particle sizes of the Ag-NPs synthesized by TDM and MWA methods were 6.08 nm and 8.91 nm, respectively.

There is a clear difference between the particle sizes calculated by XRD and TEM measurements. This is because transmission electron microscopy (TEM) visualizes the entire particle. In this case, the measured size includes the metal core of the nanoparticles, as well as any materials (e.g., reducers or stabilizers) in their outer layers. In contrast, XRD measures only the crystallite size.

Furthermore, the Debye–Scherrer formula is applicable to particles of a near-spherical shape. The discrepancy between the diameter difference values obtained from XRD patterns and those obtained from TEM observations is due to the limitations of the formula.

In the MWA method, the inherent effects of microwaves cause the nanoparticles to have different morphologies and sizes compared to the TDM. The rapid and homogeneous heating of microwaves ensures the rapid and homogeneous heating of reactants, solvents, intermediates, and products. Heating by microwaves accelerates the reduction of metals and the nucleation of the metal cluster, resulting in the formation of monodisperse nanostructures. Despite the homogeneous formation of particles through microwave heating, aggregation of nanoparticles can occur due to the inability to completely control both the intensity and extent of overheating during microwave processing. Therefore, in the TEM and XRD results, the average particle sizes obtained with the MWA method are larger than those obtained with the TDM method.

In the present study, synthesized Ag-NPs were centrifuged and washed 3 times with deionized water. The purpose of this process is to remove unreacted AgNO_3_ in the nanoparticles with deionized water. We proved that AgNO_3_ was completely separated from the solution by measuring the conductivity of the washing solution. Conductivity measurements performed in all 3 washes are shown in Table 1. Based on these results ICP-MS analysis was performed to determine the amount of Ag in Ag-NPs.

The conductivity values measured after the third washing were almost the same as the conductivity values of deionized water. These values showed that AgNO_3_, which did not react during Ag-NPs synthesis, was completely separated from the solution by washing.

ICP-MS was used to determine the amount of Ag in Ag-NPs (TDM and MWA). We confirmed that Ag^+^ ions from unreacted AgNO_3_ were completely removed by washing and conductivity measurement. Ag-NPs, which were dried and weighed, were dissolved in 5% HNO_3_ prepared from 55% HNO_3_. Dilute solutions of Ag-NPs were prepared and analyzed by ICP-MS. The amounts of Ag-NPs synthesized by TDM and MWA methods measured in ICP-MS were 0.430 mg/mL and 0.115 mg/mL, respectively. Cytotoxicity studies of Ag-NPs were performed with the values measured by ICP-MS.

Sumac-derived Ag-NPs have garnered interest because of their antimicrobial properties [48]. In its natural state, sumac possesses tannins and other phenolic chemicals that have antibacterial effects [49]. Chemical compounds derived from this plant, particularly from its fruit and leaf components, can be utilized in the production of Ag-NPs [50,51]. The antibacterial properties of Ag-NPs are mostly attributed to their capacity to attach to the bacterial cell wall, thereby disturbing the integrity of the cell membrane [52]. They also interact with intracellular enzymes to block metabolic activities and induce oxidative stress, causing damage to cells. Ag-NPs synthesized using the synergistic properties of plant components have the potential for a wider range of antibacterial activity and relatively reduced toxicity [53]. Significant enhancement in the antibacterial effectiveness of Ag-NPs synthesized with SMC was detected in our investigation. Agar diffusion and minimum inhibition tests were used to assess the enhancement in antimicrobial effectiveness against infection agents including *S*. *aureus, S*. *pneumoniae*, *E*. *coli*, *P*. *aeruginosa*, *E*. *faecalis*, and *C*. *albicans*. Ag-NPs and SMCs derived from sumac are more effective against Gram-positive bacteria such as *S*. *aureus* and *S*. *pneumoniae* and yeast such as *C*. *albicans* compared to Gram-negative pathogens such as *E*. *coli*, *P*. *aeruginosa*, and *E*. *faecalis*. Minimum inhibition concentration (MIC), minimum bactericidal concentration (MBC), and minimum fungicidal concentration (MFC) assays of SMC and Ag-NPs are represented in Table 2. The MIC and MBC values show that SMCs and Ag-NPs are effective against bacteria. The nanoparticles synthesized with MWA were effective against *S*. *aureus* (MIC = 12.5 mg/mL and MBC = 25 mg/mL) and *S*. *pneumonia* (MIC = 12.5 mg/mL and MBC = 12.5 mg/mL), while the nanoparticles synthesized with 2 mM AgNO_3_ (TDM) were effective against *E*. *coli* (MIC = 12.5 mg/mL and MBC = 25 mg/mL) and *C*. *albicans* (MIC = 12.5 mg/mL and MFC = 25 mg/mL). SMC had the same effect as standard ciprofloxacin against *S*. *pneumoniae* (MIC = 6.25 mg/mL and MBC = 12.5 mg/mL) and *E*. *coli* (MIC = 6.25 mg/mL and MBC = 12.5 mg/mL).

The antimicrobial activities are presented in Table 3. Ciprofloxacin and fluconazole were used as positive controls for bacteria and yeast, respectively. In vitro antimicrobial activity data shows that the antimicrobial activity of Ag-NPs higher than that of the SMC. Microwave synthesis of Ag-NPs is a rapid, effective, and energy-efficient technique [54]. Ag-NPs synthesized by the MWA method were more effective against bacteria than the TDM method and SMC alone. Also, MWA Ag-NPs were most effective against *S*. *pneumoniae* and *P*. *aeruginosa*. All nanoparticles were effective against *C*. *albicans*, especially MWA Ag-NPs, which had the same activity as standard fluconazole.

In MWA synthesis, the fast progression of the reaction may not always allow for exact manipulation of particle size and form. This can result in a wide range of particle sizes [55]. Exposure to high-power microwave radiation can result in overheating, causing nanoparticles to aggregate and thus impair their release when used in pharmaceutical formulations [56]. However, the ecological friendliness and cost effectiveness of these biological synthesis techniques make them worth pursuing.

Further research is being conducted to investigate the antibacterial effectiveness of Ag-NPs against resistant bacterial strains, which may provide new treatment approaches in the future.

The aim of the study was to determine the anticancer effects of Ag-NPs obtained by 2 different methods (TDM and MWA) on the human colon cancer cell line (HT-29). Furthermore, we determined the dose that does not affect healthy cells by applying different concentrations (6.25, 12.5, 25, 50, and 100 μg/mL) to healthy mouse fibroblast cells (L929).

**Figure 11:**
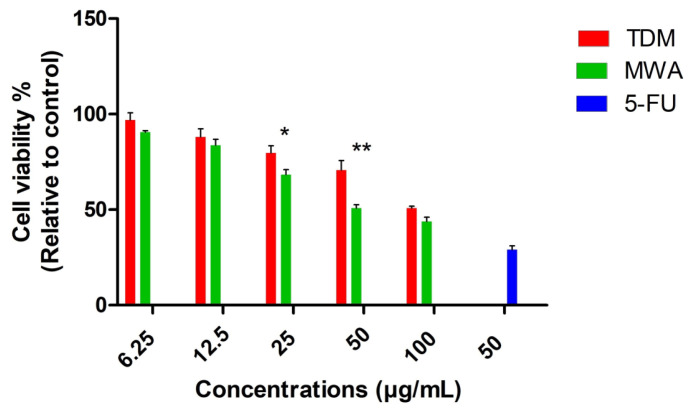
Comparison of cell viability rates in HT-29 cancer cells 48 h after treatment with TDM and MWA Ag-NPs and 5-FU.

After 48 h of treatment, the cell viability percentages obtained in HT-29 cells for all nanoparticle concentrations and the 50 μg/mL dose of 5-FU are shown in Figure 11. In this context, it is demonstrated that the Ag-NPs (MWA) sample exhibits significantly higher anticancer activity at 25* and 50** μg/mL concentrations compared to the Ag-NPs (TDM) sample (*; p < 0.05, **; p < 0.01). The evaluated nanoparticle concentrations have higher IC_50_ values compared to 5-FU (50 μg/mL).

**Figure 12:**
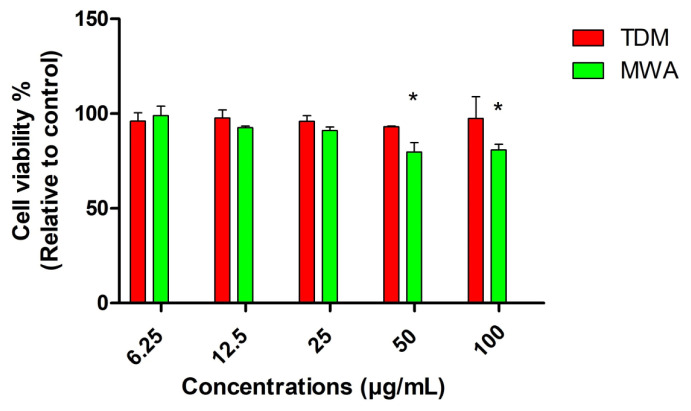
Comparison of cell viability rates in healthy L929 cells 48 h after treatment with TDM and MWA Ag-NPs.

The cell viability values obtained for L929 cells at all doses after 48 h are given in Figure 12. Cell viability was >70% at all doses, indicating no cytotoxicity for healthy cells. However, MWA Ag-NPs still had significantly higher toxicity compared to TDM Ag-NPs at 50 and 100 μg/mL doses (p < 0.05). Additionally, the inhibition observed in HT-29 cancer cells at 12.5, 25, 50, and 100 μg/mL doses was not observed in L929 healthy fibroblast cells, indicating selective inhibition at these doses. The mean IC_50_ (SE) values for the TDM and MWA Ag-NPs after 48 h were 102 μg/mL (0.32) and 48 μg/mL (0.12), respectively.

The structural properties, anticancer, and antimicrobial activities of Ag-NPs synthesized using SMC are presented in Table 4. These findings suggest that the biological applications of Ag-NPs derived from SMC are limited. Furthermore, there are few publications involving cancer cells, and this study is the first to investigate their effects on HT-29 cancer cells.

## 4. Conclusion

In this study, we prepared Ag-NPs using the extract of sumac fruits using TDM and MWA methods. Sumac fruits are rich in biological agents such as fatty acids, flavonoids, saponins, phenolics, and volatile compounds that are effective in the reduction of silver ions and stabilization of nanoparticles. Although nanoparticles were synthesized by both methods without the addition of any reducing chemicals in a nontoxic, environmentally friendly, and low-cost manner, the MWA method was a simpler, safer, and faster than the TDM.

The obtained Ag-NPs were characterized by FT-IR, UV–Vis, SEM/EDX, TEM, XRD, ICP-MS, and conductivity measurement. Collectively, the data show that the Ag-NPs had a spherical morphological structure, and maximum absorbance peaks were observed in the 433 and 436 nm SPR band. The sizes of Ag-NPs synthesized by the MWA method were larger than the nanoparticles synthesized by TDM. XRD results showed good agreement with the face-centered cubic crystal structure of Ag-NPs synthesized by TDM and MWA methods with crystallite sizes of 6.08 nm and 8.91 nm, respectively. XRD and TEM analysis showed that the nanoparticles synthesized by MWA had a more uniform and homogeneous distribution.

Ag-NPs synthesized by the MWA method were more effective against bacteria than TDM Ag-NPs and SMC. Also, MWA Ag-NPs were most effective against *S*. *pneumoniae* and *P*. *aeruginosa*. The results indicated that all nanoparticles showed activity against *C*. *albicans*. In particular, MWA Ag-NPs were as effective against *C*. *albicans* than the reference standard fluconazole.

For the first time, cell viability assays showed that Ag-NPs inhibited the proliferation of HT-29 cells without toxicity to healthy control fibroblast cells (L929) in comparison to 5-FU (50 μg/mL). This means that the green synthesized nanoparticles made using 2 different techniques had specific anticancer properties against intestinal cancer cells.

These results show that MWA synthesis is an economical, effective, and fast way to obtain Ag-NPs. In the future, the nanoparticles obtained by green synthesis can be used in the large-scale production of many fields of application. However, there are still some difficulties in ensuring the homogeneous size distribution of nanoparticles obtained by TDMs.

The MWA method will be used in future studies. Our findings show that it is one of the most suitable methods for green synthesis of nanoparticles from plant extracts. In addition, there is significant potential for the development of various biological applications of nanoparticles synthesized with SMC both in vitro and in vivo.

## Figures and Tables

**Table 1:** Conductivity measurements of Ag-NP solutions*.

Ag-NPs reaction solution	Sample 1 (μS/cm)	Sample 2 (μS/cm)	Sample 3 (μS/cm)
First filtrate	95	112	244
1^st^ wash	22	24	20
2^nd^ wash	9	17	20
3^rd^ wash	6	9	7

* Deionized water: 6 μS/cm

Sample 1: 1mM AgNO_3_-15 mL SMC pH=3.5

Sample 2: 1mM AgNO_3_-15 mL SMC pH=7

Sample 3: 1mM AgNO_3_-15 mL SMC pH=9

**Table 2:** MIC, MBC, and MFC assays of SMC and Ag-NPs.

Sumac extract and Ag-NPs	Microorganism / MIC, MBC, and MFC (mg/mL)					
	*S. aureus*	*S. pneumoniae*	*E. coli*	*P. aeruginosa*	*E. faecalis*	*C. albicans*
	MIC, MBC	MIC, MBC	MIC, MBC	MIC, MBC	MIC, MBC	MIC, MFC
Ag-NPs (TDM, 1 mM)	100, 100	25, 50	50, 100	100, 100	100, 100	25, 25
Ag-NPs (TDM, 2 mM)	50, 100	12.5, 25	12.5, 25	100, 100	100, 100	12.5, 25
Ag-NPs (MWA, 1 mM)	12.5, 25	12.5, 12.5	25, 50	50, 100	100, 100	25, 50
Sumac	12.5, 12.5	6.25,12.5	6.25, 12.5	100, 100	12.5, 25	12.5, 25
Control*	3.12	6.25	6.25	6.25	6.25	6.25

* Control: ciprofloxacin (for bacteria) and fluconazole (for yeast).

**Table 3:** In vitro antimicrobial activity of SMC and Ag-NPs by agar well diffusion.

Sumac extract and Ag-NPs	Microorganism/antimicrobial activity (zone of diameter, mm)					
	*S. aureus*	*S. pneumoniae*	*E. coli*	*P. aeruginosa*	*E. faecalis*	*C. albicans*
Ag-NPs (TDM, 1 mM)	6 ± 0.18	7 ± 0.12	6 ± 0.10	8 ± 0.42	8 ± 0.45	10 ± 0.60
Ag-NPs (TDM, 2 mM)	9 ± 0.32	9 ± 0.17	7 ± 0.5	9 ± 0.11	9 ± 0.10	10 ± 0.55
Ag-NPs (MWA, 1 mM)	8 ± 0.01	11 ± 0.20	9 ± 0.05	11 ± 0.50	10 ± 0.35	12 ± 0.18
Sumac	7 ± 0.25	9 ± 0.12	7 ± 0.24	10± 0.60	8 ± 0.15	9 ± 0.30
Control*	16 ± 0.05	12 ± 0.22	10 ± 0.45	10 ± 0.67	11 ± 0.24	12 ± 0.40

* Control: ciprofloxacin (for bacteria) and fluconazole (for yeast).

**Table 4:** A summary of the structural properties, anticancer, and antimicrobial activities of Ag-NPs synthesized using SMC.

AgNO_3_ Concentration Ag-NPs Size (nm), morphology reaction time, temperature/condition	SPR band λ_max,_ (nm)	Anticancer Cell* Dose, Cell Viability%	IC_50_ (μg/ mL)	Antimicrobial Activity	Reference
0.5 mM 19.81 ± 3.67, spherical 60 min, 35 C	438	HepG2 4.68 μM, 5.73%	---	*E. coli*	[41]
1 mM 4, spherical 48 h, 75 C	400	---	---	*B. cereus, B. subtilis* *E. faecalis, P. aeruginosa, C. albicans*	[48]
3 mM 10–25, spherical Stirred at 150 rpm for 30 min Incubated under dark condition 24 h, RT	417–420	MCF-7 160 μg/mL, 12.5%, HFF-2 160 μg/mL, 19.2%	(24–48 h) MCF-7: 14.27–13.4 HFF-2: 62.7–81.73	---	[57]
100 mM 43.82–39.55, spherical 10–20 min, RT	276	---	---	*S. aureus, A. baumannii* *E. faecalis, P. aeruginosa*	[58]
1 mM 30–100, spherical Manually stirring, placed in the dark	420	---	---	*E. faecalis, S. mutans* *S. sobrinus, L. acidophilus* *S. salivarius*	[59]
1 mM 22, spherical 24 h, RT	433	HT-29, 100 μg/mL, 48.01% L929, (All values > 70%)	102 ± 0.32 (48 h)	*S. aureus, S. pneumonia* *E. coli, P. aeruginosa* *E. faecalis, C. albicans*	Current study (TDM)
1 mM 41.85, spherical 5 min, 800 W	436	HT-29 50 μg/mL, 48.01%; 100 μg/mL, 44.6% L929 (All values > 70%)	48 ± 0.12 (48 h)	*S. aureus, S. pneumonia* *E. coli, P. aeruginosa* *E. faecalis, C. albicans*	Current study (MWA)
